# Unraveling the Structure and Dynamics of Ac-PHF6-NH_2_ Tau Segment Oligomers

**DOI:** 10.1021/acschemneuro.4c00404

**Published:** 2024-08-31

**Authors:** Iuliia Stroganova, Zenon Toprakcioglu, Hannah Willenberg, Tuomas P. J. Knowles, Anouk M. Rijs

**Affiliations:** † Division of Bioanalytical Chemistry, Department of Chemistry and Pharmaceutical Sciences, Amsterdam Institute of Molecular and Life Sciences, Vrije Universiteit Amsterdam, De Boelelaan 1105, 1081 HV Amsterdam, The Netherlands; ‡ Centre for Analytical Sciences Amsterdam, 1098 XH Amsterdam, The Netherlands; § Centre for Misfolding Diseases, Yusuf Hamied Department of Chemistry, University of Cambridge, Cambridge CB2 1EW, U.K.; ∥ Cavendish Laboratory, Department of Physics, University of Cambridge, Cambridge CB3 0HE, U.K.

**Keywords:** peptide aggregation, ion mobility mass spectrometry, amyloid oligomers, tau, kinetics, Alzheimer’s disease

## Abstract

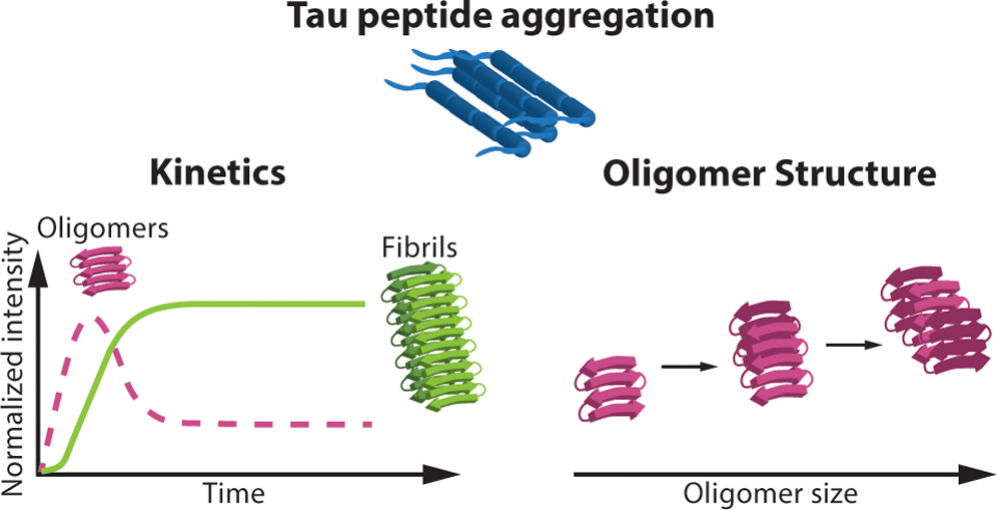

The aggregation of the proteins tau and amyloid-β is a salient feature of Alzheimer’s disease, the most common form of neurodegenerative disorders. Upon aggregation, proteins transition from their soluble, monomeric, and functional state into insoluble, fibrillar deposits through a complex process involving a variety of intermediate species of different morphologies, including monomers, toxic oligomers, and insoluble fibrils. To control and direct peptide aggregation, a complete characterization of all species present and an understanding of the molecular processes along the aggregation pathway are essential. However, this is extremely challenging due to the transient nature of oligomers and the complexity of the reaction networks. Therefore, we have employed a combined approach that allows us to probe the structure and kinetics of oligomeric species, following them over time as they form fibrillar structures. Targeting the tau protein peptide segment Ac-PHF6-NH_2_, which is crucial for the aggregation of the full protein, soft nano-electrospray ionization combined with ion mobility mass spectrometry has been employed to study the kinetics of heparin-induced intact oligomer formation. The oligomers are identified and characterized using high-resolution ion mobility mass spectrometry, demonstrating that the addition of heparin does not alter the structure of the oligomeric species. The kinetics of fibril formation is monitored through a Thioflavin T fluorescence assay. Global fitting of the kinetic data indicates that secondary nucleation plays a key role in the aggregation of the Ac-PHF6-NH_2_ tau segment, while the primary nucleation rate is greatly accelerated by heparin.

Aggregation of peptides and proteins from soluble monomers into insoluble fibrils is associated with several neurodegenerative disorders, including Alzheimer’s and Parkinson’s diseases.^1^ Tau is an intrinsically disordered protein that stabilizes microtubules,^2^ however abnormal tau phosphorylation causes the loss of its function and the formation of neurofibrillary tangles (NFTs).^3^ Diseases associated with NFT formation are summarized as tauopathies, including Alzheimer’s disease, frontotemporal dementia, Pick’s disease and progressive supranuclear palsy.^4,5^ Several studies indicate that the highly heterogeneous oligomeric intermediates formed during tau assembly are more toxic than the NFTs themselves.^6−8^ Heparin, together with other anionic factors, such as RNA,^9,10^ is known to accelerate the typically slow tau assembly in vitro. This enhancement is related to overcoming of the electrostatic repulsion of the positively charged tau protein when negatively charged agents are introduced.^11^

Protein aggregation is a very complex reaction in which several molecular processes occur simultaneously.^12,13^ Primary processes include primary nucleation and elongation, where monomers associate to form a nucleus and where these monomers are added to the ends of the protofibrils to facilitate their growth, respectively. These structures can serve as nucleation sites during surface-catalyzed secondary nucleation to grow new nuclei. Fibril fragmentation is another secondary process that can contribute significantly to the protein aggregation. Monitoring the aggregation kinetics can provide insight into the interplay between these different microscopic processes that occur during protein aggregation and allows to identify which process is predominant. For example, several studies have shown that secondary processes play an important role in tau aggregation,^14−16^ while its primary nucleation is slow. To gain further insight into the aggregation kinetics, it is essential to separately examine the different types of species, i.e., monomers, oligomers, and fibrils, that appear during aggregation, even though they are present concurrently. The measurement of fibril aggregation kinetics is a well-established bulk technique, based on fluorescent dyes. One such dye is Thioflavin T (ThT), which binds to fibrils after a significant amount of β-sheets have been formed.^17^ ThT fluorescence assay, combined with the web-based software AmyloFit^18^ developed by the Knowles group, allows the determination and verification of a molecular mechanism for aggregation reactions through the global fitting of kinetic data for fibrillar species.^19−21^ Studies focusing on the time-dependent structural properties of early-stage oligomers come with significant challenges. These difficulties arise from the intrinsic properties of the oligomers themselves. Based on the kinetic modeling of many amyloidogenic systems, such as Aβ40, Aβ42, α-synuclein, and tau, the oligomeric species were found to be low in abundance and transient.^22^ These oligomers can coexist in several populations, that can interconvert, either grow into full fibrils or dissociate back into monomers very rapidly,^22^ making the study of these transient oligomers a challenging task. Methods to study these oligomers are currently under active development. For instance, the Klenerman group developed a single-molecule Förster resonance energy transfer (smFRET) assay to characterize oligomers of different amyloidogenic systems, including tau.^23^ They studied the effect of mutations in the K18 tau construct, which contains all four tau repeats, on the kinetics of aggregation. Kjaergaard et al. later extended this smFRET method to account for electrostatic interactions between the mutant K18 tau segment and heparin, using high ionic strength buffers to distinguish between two populations of oligomers.^24^ This study showed that heparin directly contributes to the formation of early-stage oligomers. A more stable oligomer population, presumably stabilized by hydrophobic interactions, did not lead to fibril formation. Another technique that allows to separate individual oligomeric species and can provide dynamic information is electrospray ionization mass spectrometry (ESI-MS)^25^ When mass spectrometry is combined with techniques, such as ion mobility spectrometry (IMS),^26−29^ hydrogen–deuterium exchange,^30^ cross-linking,^31^ or infrared action spectroscopy,^32,33^ structural information on individual oligomeric species along the tau aggregation pathway can be obtained. For example, Larini et al.^28^ showed that heparin accelerated aggregation of tau peptide 273–284 from the second repeat (R2) using ion mobility mass spectrometry (IM-MS). They proposed an aggregation mechanism by using molecular dynamics simulations where heparin binds to extended conformations and promotes their subsequent assembly into fibrils. Ganguly et al.^29^ showed that tau peptide 306-317 from the third repeat of tau (R3) aggregates rapidly in the presence of heparin. Rodriguez Camargo et al.^14^ focused on the spontaneous self-assembly of the tau 304–380 peptide segment in sodium phosphate buffer using size exclusion chromatography combined with isotope-labeled MS and tryptic digestion to elucidate the origin of the oligomers. Their results revealed that the tau oligomers originate from the secondary nucleation process. However, no information on the time evolution of individual oligomers by (IM)-MS has been reported to date to our knowledge.

Here, we study the Ac-PHF6-NH_2_ peptide segment (^306^VQIVYK^311^) of the tau protein, which is located in the third repeat (R3) of tau and is part of the aggregation core of the protein.^34^ The main focus of this study is to gain insights into the structure and dynamics of individual, intact oligomers of the Ac-PHF6-NH_2_ peptide in their native state and how this correlates with the full picture of the aggregation process, i.e., bulk measurements of amyloid fibrils. In addition, the effect of heparin on the structure of the oligomers and the aggregation mechanism is investigated. Therefore, we have designed a holistic approach by using soft nano-ESI and ion mobility mass spectrometry (IM-MS) together with bulk methods, such as ThT fluorescence assays, circular dichroism (CD) spectroscopy, and transmission electron microscopy (TEM), see Figure S1 in the Supporting Information (SI). IM-MS is employed to separate and characterize the structure of individual, intact Ac-PHF6-NH_2_ oligomers over time. The ThT assays together with global fitting of kinetic data are used to elucidate the dominant molecular mechanism of aggregation of the Ac-PHF6-NH_2_ peptide in the presence of heparin and its role in tau aggregation. It has previously been shown that the disease-relevant structures of tau are formed in a nonvolatile buffer, such as sodium phosphate buffer,^35^ which cannot be used directly with our IM-MS approach. Therefore, we used ammonium acetate (AA) solution with heparin for all the methods reported in this paper. Additionally, TEM allows us to determine the fibrillar morphology, while CD spectroscopy provides secondary structure information regarding the presence of β-sheet content.

## Results

### Monitoring the Aggregation Kinetics of Ac-PHF6-NH_2_ Peptide Using ThT Assays

**Figure 1:**
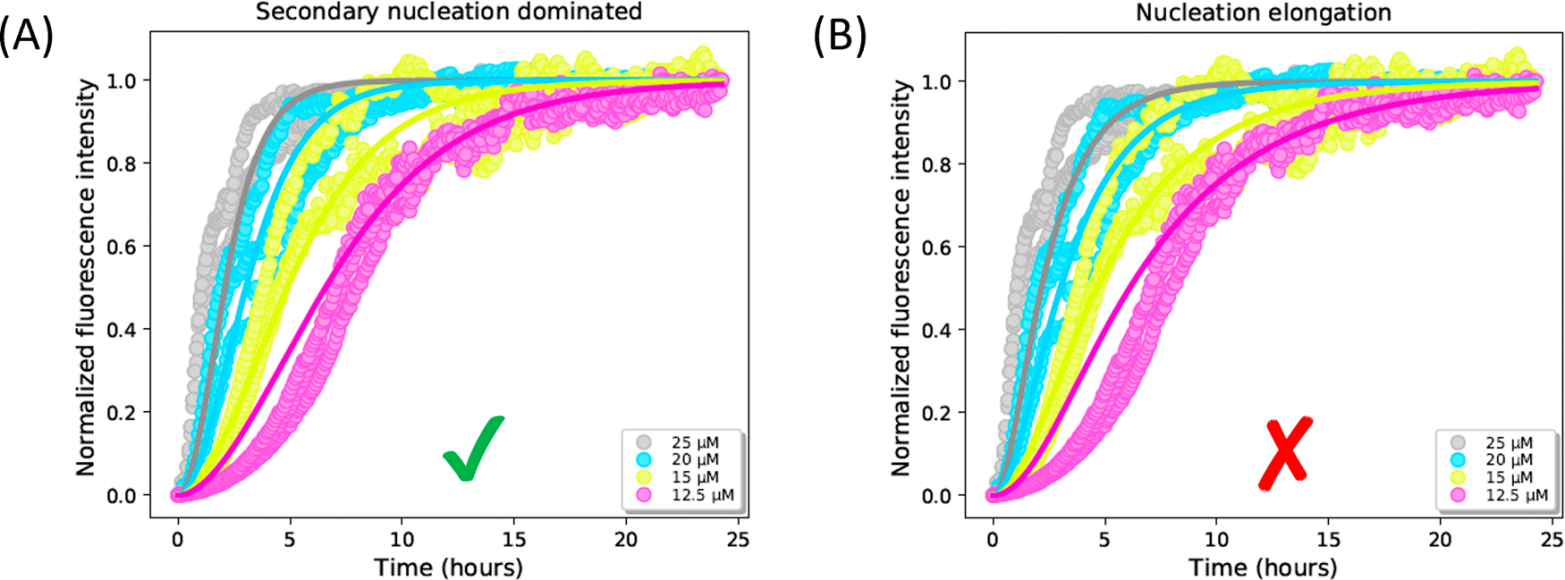
Aggregation kinetics of Ac-PHF6-NH_2_ peptide in 10 mM AA with 20 μM ThT and 1.5 μM heparin at peptide concentrations of 25, 20, 15, and 12.5 μM (gray, blue, yellow, and pink, respectively). Each peptide concentration was measured in triplicate. (A) The data are fitted (solid lines) using a global fit based on the secondary nucleation-dominated model in AmyloFit. The rate constants are *k*_+_*k*_*n*_ = 1.03 × 10^6^ M^–3^ s^–2^, *k*_+_*k*_2_ = 1.98 × 10^11^ M^–4^ s^–2^ with *n*_*c*_ = *n*_2_ = 3, and the mean residual error (MRE) is 0.00543. (B) The data are fitted (solid lines) using the nucleation elongation model in AmyloFit, resulting in the rate constants *k*_+_*k*_*n*_ = 1.03 × 10^6^ M^–3^ s^–2^ with *n*_*c*_ = 3, and the mean residual error (MRE) is 0.00571.

The self-assembly of the Ac-PHF6-NH_2_ peptide was monitored using ThT fluorescence. Without the addition of heparin, the aggregation of the Ac-PHF6-NH_2_ peptide is very slow and the first increase in ThT fluorescence appears only after 1 week.^36^ The aggregation process in the current study is initiated by the addition of heparin in a controlled manner, which greatly accelerates the process. The aggregation kinetics obtained at four different peptide concentrations with heparin are presented in Figure 1. The kinetic data show that the aggregation of Ac-PHF6-NH_2_ is accelerated with increasing peptide concentration. After about 1 day, all concentrations have reached the plateau phase, indicating that the peptides self-assembled into their fibrillar state.

To determine the microscopic processes underlying the aggregation of the Ac-PHF6-NH_2_ peptide, the web-based AmyloFit software was used.^18^ This platform uses quantitative kinetic assays and global fitting to establish and validate a molecular mechanism for aggregation reactions that matches experimental kinetic data. All models feature primary nucleation and elongation, while some models also include fragmentation and/or secondary nucleation. Fragmentation models were not considered because no agitation was applied in our experiments. Among these aggregation models, the secondary nucleation model fits our data best and gives the lowest mean residual error (MRE) of 0.00543, see Figure 1A. The models with more complex saturating elongation and secondary nucleation processes were also evaluated (see Figure S2 in the Supporting Information). These models, presented in Figure S2C,D, show an adequate fit for the lowest peptide concentration of 12.5 μM at times 0–10 h, however, they deviate from the experimental data at longer time points (10–25 h). Moreover, these models have higher MREs (0.00730 and 0.00729 respectively) than the secondary nucleation model (0.00543). Therefore, the secondary nucleation model is an overall better fit to our data than these two models. Previous studies on the Ac-PHF6-NH_2_ peptide demonstrated that it follows a seeded nucleation-elongation mechanism when buffers with a high salt concentration were used instead of heparin.^37,38^ The nucleation elongation model was also considered for our data, but showed a poorer fit, especially for the lowest peptide concentration of 12.5 μM from 0 to 7 h (see Figure 1B). This also indicates that the addition of heparin possibly favors a different process than a high salt concentration.

Using the secondary nucleation dominated model, the combined microscopic rate constants were obtained as global fit parameters. The following rate constants were derived: *k*_+_*k*_*n*_ = 1.03 × 10^6^ M^–3^ s^–2^ and *k*_+_*k*_2_ = 1.98 × 10^11^ M^–4^ s^–2^ with *n*_*c*_ = *n*_2_ = 3, where *k*_*n*_, *k*_+_, and *k*_2_ are the primary nucleation rate, the elongation rate, and the secondary nucleation rate constants, respectively, and *n*_*c*_ and *n*_2_ are the primary and secondary nucleus sizes, respectively. Typical values for the aggregation of Aβ42 are *k*_+_*k*_*n*_ = 900 M^–2^ s^–2^; *k*_+_*k*_2_ = 4 × 10^10^ M^–3^ s^–2^ with *n*_*c*_ = *n*_2_ = 2.^19^ The ratio of secondary to primary nucleation rates (*k*_2_/*k*_*n*_) in our case is 10^5^, meaning that secondary nucleation is more dominant than primary nucleation. This ratio is also rather high (∼10^7^) for Aβ42, which similarly follows a secondary nucleation mechanism.^19^ Since individual rate constants cannot be derived from our data, it can only be assumed that elongation and secondary nucleation rates are much higher than the primary nucleation. As previously discussed by Arosio et al., high elongation and secondary nucleation rates significantly reduce the length of the lag phase,^12^ which is in good agreement with the shape of our kinetic curves that show a very short lag phase.

Pretti et al.^11^ used coarse-grained simulations to show that heparin acts as a nucleation site for aggregation of the Ac-PHF6-NH_2_ peptide while remaining attached to the elongated fibrils. The electrostatic interaction between the negatively charged heparin and the positively charged lysine side chain of the Ac-PHF6-NH_2_ peptide has a strong effect in enhancing the ability of this peptide to nucleate. This can explain a rather rapid aggregation process. Although these interactions with heparin cannot be taken into account in AmyloFit, the fitting of kinetic data clearly shows that secondary nucleation plays an important role in the aggregation of the Ac-PHF6-NH_2_ peptide in the presence of heparin. Heparin not only facilitates the nucleation of monomers but also potentially enhances elongation as the fibrillar aggregates remain attached to the anionic polyelectrolyte, as shown in simulations.^11^

### CD Spectra Show the β-Sheet Formation

**Figure 2:**
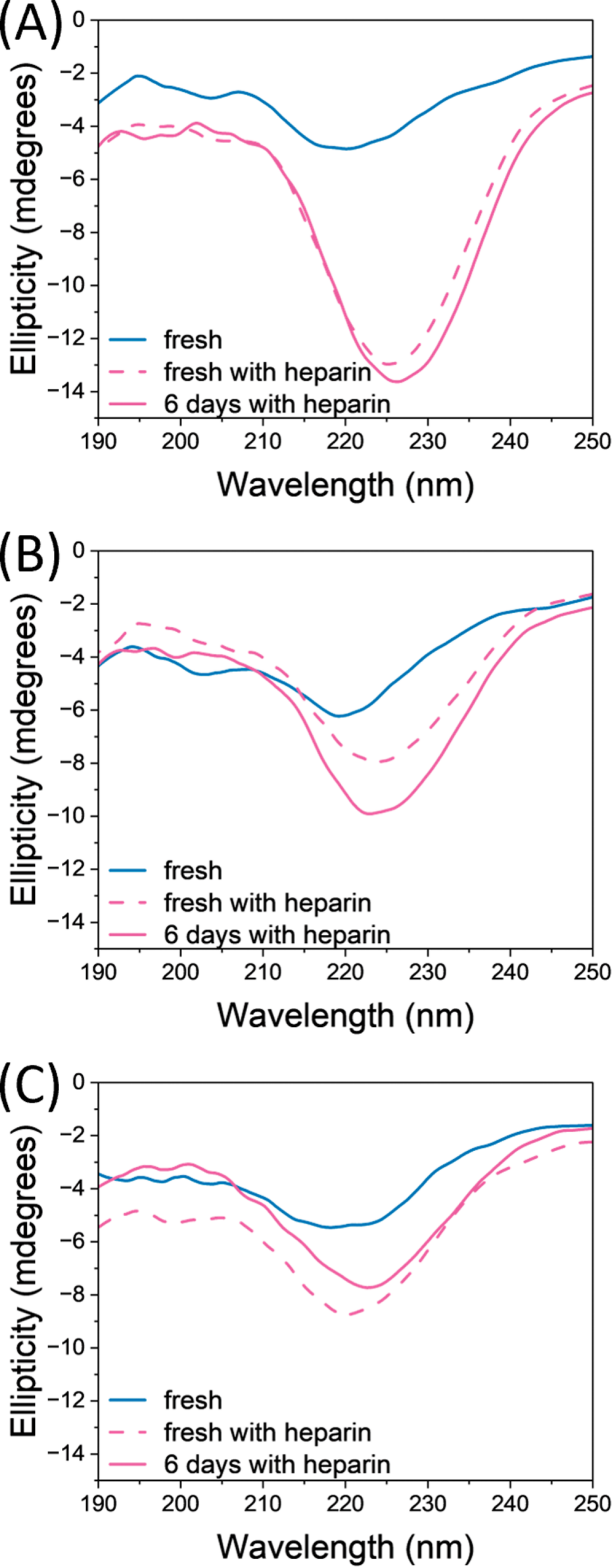
Circular dichroism (CD) spectra of Ac-PHF6-NH_2_ peptide in 10 mM AA at peptide concentrations: (A) 100 μM, (B) 75 μM, and (C) 50 μM. The blue line shows freshly prepared peptide sample without heparin. Samples prepared with 1.5 μM heparin are shown in pink, where the dashed line shows a freshly prepared sample and the solid line corresponds to a sample incubated at room temperature for 6 days.

Figure 2 shows CD spectra of the Ac-PHF6-NH_2_ peptide at three different peptide concentrations (100, 75, and 50 μM in 10 mM AA). Higher peptide concentrations are used for CD than for the ThT assays to obtain an optimal signal-to-noise ratio. The CD spectra of freshly prepared monomeric peptide solutions without heparin (blue lines) show a negative band at 220 nm. Arya et al.^39^ observed a similar spectral shape for monomeric solutions without heparin, which they assigned to a random coil. As previously shown by gas-phase infrared spectroscopy, a PHF6 dimer is organized into a β-sheet,^32^ which can explain that there is a feature at approximately 220 nm corresponding to β-sheet in a fresh monomeric solution.

Immediately after the addition of heparin, the minimum in the spectra redshifts from 220 nm to approximately 225 nm (dashed pink lines), indicating an increase in β-sheet content. This is consistent with the ThT assays showing that the Ac-PHF6-NH_2_ peptide aggregates almost immediately at these elevated peptide concentrations (compared to our MS or ThT assay experiments). The position of the negative bands around 220–230 nm is slightly redshifted with respect to typical β-sheet signatures (∼218 nm). A similar shift has been observed previously for tyrosine containing cyclic peptides where the peak position was effected due to the absorption of the aromatic side chain.^40^ After 6 days of incubation of the Ac-PHF6-NH_2_ peptide solutions with heparin at room temperature, no significant changes in the spectral shape were observed (solid pink lines). This suggests that the peptide conformation in a solution is still very dynamic and not all peptides are completely converted into fully grown organized fibrils. However, after about one month of incubation with heparin at room temperature, a positive band at about 200 nm and a negative peak at about 220 nm were observed in the CD spectrum (see Figure S3 in the SI). These are the typical β-sheet signatures, that clearly indicate the presence of mature fibrils with fully organized β-sheet structures for the Ac-PHF6-NH_2_ peptide.

### TEM Imaging Displays the Morphology of Aggregates

**Figure 3:**
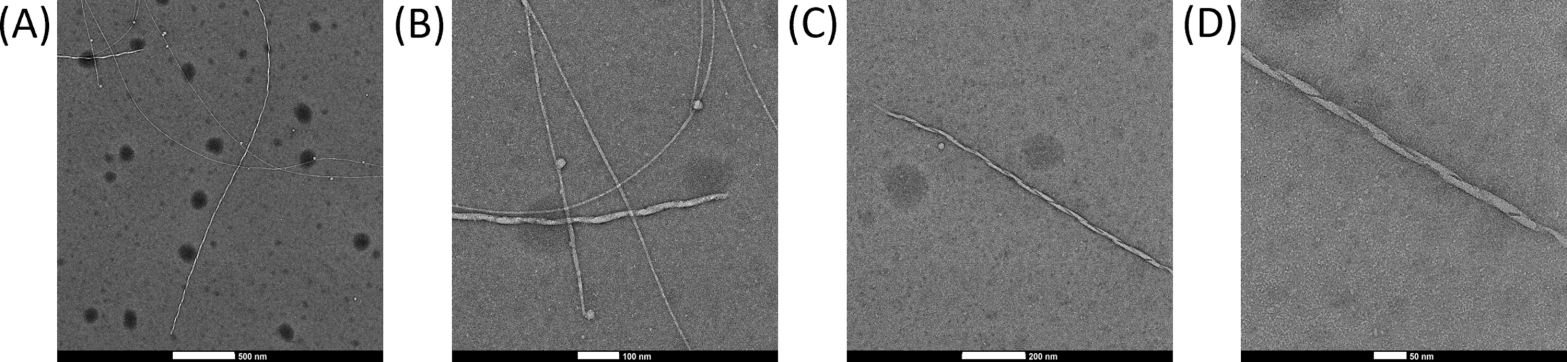
TEM images of Ac-PHF6-NH_2_ peptide 25 μM in 10 mM AA with 1.5 μM heparin and 20 μM ThT from the well plate visualized after 4 days. The scale bar is 500 nm in (A), 100 nm in (B), 200 nm in (C), and 50 nm in (D).

Transmission electron microscopy (TEM) was used to visualize fibrillar structures. The peptide sample was removed from the well plate and placed on the TEM grid for imaging 4 days after its preparation. At this time point, the ThT assays have already reached the plateau, and therefore the saturation of ThT binding to β-sheet fibrillar structures coinciding with the CD experiments, which showed the β-sheet signatures. The Ac-PHF6-NH_2_ peptide forms long fibrils as shown in Figure 3A. Figure 3B shows that some of the fibrils are straight, while others are twisted, as was previously observed under highly aggregated conditions, i.e., high peptide and salt concentrations.^41^Figure 3C shows a single twisted fibril, which reveals a ribbon-like structure when zoomed in (Figure 3D). Arya et al.^39^ showed that the Ac-PHF6-NH_2_ peptide forms predominantly twisted fibrils in the presence of heparin compared to the straight filaments formed upon addition of 150 mM NaCl. In our case, both twisted and straight fibrils were observed, which may be due to the lower concentration of heparin used in our study (1.5 μM versus 15 μM). Additionally, the fibrils that are formed under our conditions seem to be less abundant, which is a result from the lower peptide and heparin concentrations, shorter incubation time, and lower incubation temperature.

### IM-MS Spectra Reveal Oligomer Dynamics and Structure

**Figure 4:**
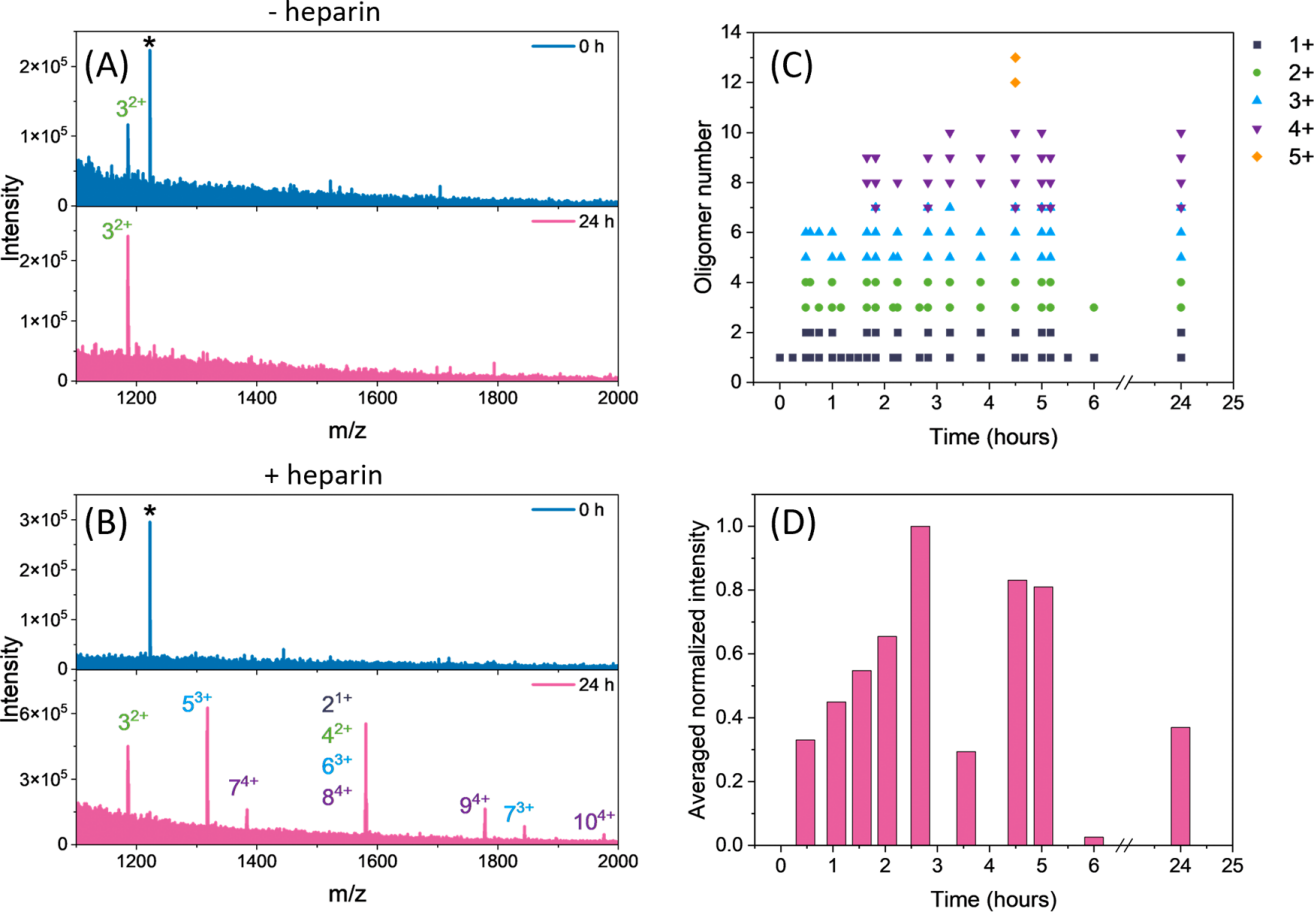
Summarized mass spectrometry data of 20 μM of Ac-PHF6-NH_2_ peptide in 10 mM AA. (A) Averaged mass spectra without heparin measured immediately after the sample preparation (blue) and after 24 h (pink). (B) Averaged mass spectra with addition of 1.5 μM heparin measured immediately after addition of heparin (blue) and after 24 h (pink). The peptide oligomers are color-coded and denoted as *n*^*z*+^, the asterisk corresponds to the TuningMix calibrant of *m*/*z* 1222. (C) Oligomer abundance versus time in the presence of heparin from five independent measurements. Each dot represents an oligomer with a specific *n*^*z*+^. (D) Averaged normalized intensity of all oligomers appearing over time in the presence of heparin.

To gain insight into the early steps of aggregation and to follow the formation of the oligomeric species of the Ac-PHF6-NH_2_ peptide, trapped ion mobility mass spectrometry (TIMS) was employed. The full mass spectra are presented in Figure S4, where the highest intensity peak corresponds to the singly charged monomer *m*/*z* 790.5. Figure 4A shows enlarged mass spectra focusing on the higher order oligomeric region,^36^ recorded immediately after the sample preparation (blue) and after 24 h (pink) without the addition of heparin, showing that no higher order oligomers appeared over time without heparin. The oligomeric species are denoted as *n*^*z*+^, where *n* is the number of monomers in the oligomer and *z* is its charge state. In contrast, a large population of oligomers of the Ac-PHF6-NH_2_ peptide is detected after 24 h of heparin addition, whereas no oligomers were present immediately after heparin addition (see Figure 4B).

To provide insight into the time scale on which these oligomers appear, we have measured the mass spectrum at approximately 30 min time intervals up to 6 h, followed by one time point after 24 h. These experiments were repeated over several days. Figure 4C illustrates which oligomers appeared over time after the addition of heparin by plotting the oligomer number *n* versus time, with the symbols indicating the variety of charge states. Ion mobility mass spectrometry was used to identify the oligomers.^36^ It can be seen that the first oligomers appear very quickly at 30 min and they continue to grow in size, with the largest oligomer of 13^5+^ present at 4.5 h. Thereafter, the detected oligomer population decreases, although a significant amount of soluble oligomers are still present at 24 h. After 3 days, the peptide sample could not be properly introduced into the mass spectrometer, indicating a significant population of insoluble protofibrils and the apparent difficulty in analyzing them by IM-MS. Figure 4D shows the normalized averaged intensity of all oligomers over time. The intensity of an oligomer was taken as the intensity of the highest peak in the corresponding isotopic distribution in the mass spectrum. All oligomer intensities from one measurement were summed per time point, normalized, and then averaged over five measurements. The total oligomer intensity increases with time, peaking at approximately 2.5 h, and then declines. It is interesting to note that the highest oligomer intensity coincides with the half time point on the kinetic curve (see Figure 1A).

**Figure 5:**
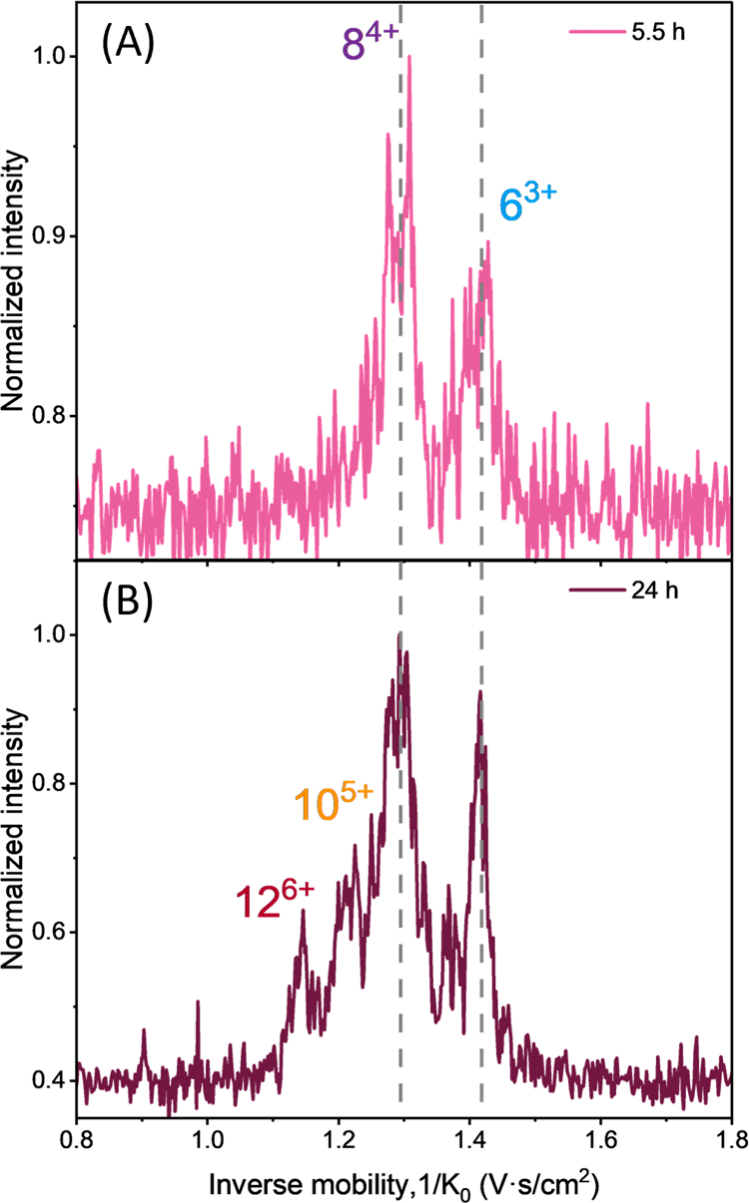
Quadrupole selected ion mobility spectra of *m*/z 1580 of 20 μM of Ac-PHF6-NH_2_ peptide in 10 mM AA with 1.5 μM heparin measured at 5.5 h (A) and at 24 h (B) after heparin addition.

To evaluate the growth of the isobaric oligomeric species with the same *m*/*z*, the IM approach with quadrupole selection is used in a similar workflow as previously presented.^36^ Here we focus on *m*/*z* 1580, corresponding to [2*n*]^*nz*+^ oligomers, where *n* is the number of monomers and *z* is the charge state. These oligomers were assigned and analyzed in our recent ESI-TIMS experiments.^36^ The oligomers of this [2*n*]^*nz*+^ mass channel start to appear after 30 min (see Figure 4C). Figure 5 shows quadrupole-selected total ion mobility spectra of *m*/*z* 1580 acquired after 5.5 h and after 24 h of heparin addition. Both ion mobility spectra contain two main peaks corresponding to the 6^3+^ and 8^4+^ oligomers. The assignment is based on the isotopic pattern of the extracted mass spectra of these two peaks (see Figure S5B). After 24 h, two additional peaks appeared in the ion mobility spectrum corresponding to the higher order oligomers, i.e., 10^5+^ and 12^6+^ (Figure 5B). The extracted mass spectra did not show an unambiguous isotopic pattern for these two mobility peaks (see Figure S5B). Therefore, we assigned these peaks to the 10^5+^ and 12^6+^ oligomers based on their reduced ion mobility values, which coincide with our previous experiments.^36^ The lower charge state oligomers (2^1+^ and 4^2+^) were not observed here due to their low intensity and the constraints of the selected ion mobility window.

To gain more insight into the structural information on the oligomers, the ion mobility values were calibrated into collision cross section (CCS) values, which allows the determination of the overall three-dimensional structural shape of the ions in the gas phase, i.e., extended vs compact conformations.^42^ The derived CCS values of the oligomers measured in the presence of heparin are the same as those previously observed in the study of Ac-PHF6-NH_2_ aggregation without heparin,^36^ see Table S1. This indicates that although heparin is known to alter the final morphology of amyloid fibrils, which is different from the disease-relevant structures,^43^ the early-stage oligomeric species appear to have the same or very similar conformation as those formed in the absence of heparin. The derived CCS values versus the oligomer number *n* are shown in Figure S6. To evaluate the growth of the peptide oligomers, an estimate of the CCS values based on isotropic growth is plotted in Figure S6 (green line). This isotropic model refers to the uniform growth in all directions, where the CCS values grow as σ_1_·*n*^2/3^ (*n* is the number of peptide monomers in the oligomer and σ_1_ is the CCS value of the monomer).^44^ The measured CCS values fall below the isotropic curve starting from the hexamer. This has been previously observed for a number of peptide oligomers^45−47^ and implies that the oligomers have a densely packed structure. In particular, in the case of human islet amyloid polypeptide (IAPP) segments, Young et al. showed that the increasing compactness of the oligomers with their increasing size is related to the formation of multi-layered stacked β-sheet structures.^47^ This multi-layered, densely packed arrangement was also previously reported by Matthes et al. for the R3 tau peptide studied using molecular dynamics simulations.^48^ The unidirectional linear fibrillar growth,^44^ indicated by the red line in Figure S6, does not overlap with the experimental data, confirming the formation of highly packed multilayers during aggregation growth.

## Discussion

**Figure 6:**
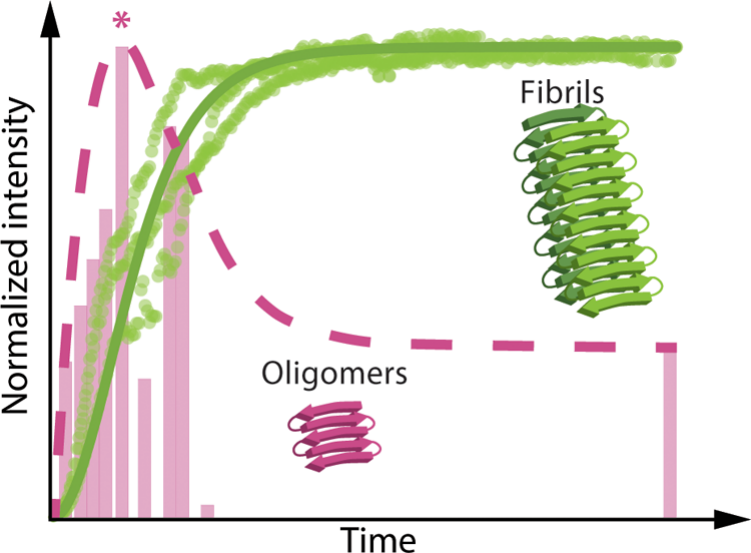
Schematic illustration of the kinetics of fibrils (green) and oligomers (pink) of Ac-PHF6-NH_2_ peptide in the presence of heparin over time. The experimental data is displayed in semi-transparent colors. The solid green line represents the fit of the kinetic data for fibrils, while the dashed pink line follows the trend of the experimental data for the oligomers.

Using the IM-MS combined with soft nano-ESI ionization, we have identified and characterized the individual oligomers of the Ac-PHF6-NH_2_ peptide that are formed during aggregation in the presence of heparin. The peptide oligomers appear very rapidly, about 30 min after the addition of heparin, coinciding with the start of the exponential growth of the fibrils, as illustrated in Figures 6 and S7. This implies that these oligomers are likely the precursors for fibril formation, i.e., they are being used for fibril formation and growth. The highest intensity of the oligomers is reached at 2.5 h, marked with a pink asterisk in Figures 6 and S7, which is close to the half time of fibrils, where they are present at 50% of their maximum concentration.^12^ At the same time, the monomers are also present at high fibril levels. Similar behavior has been observed by the Knowles group for the Aβ42 peptide, which forms oligomers in significant amounts in the presence of both monomers and fibrils.^49^ Aβ42 has shown to follow a secondary nucleation mechanism of aggregation, which coincides with our findings for the Ac-PHF6-NH_2_ peptide segment. Moreover, a recent time-resolved cryo-EM study by Lövestam et al. on the longer tau segment, which includes PHF6 peptide, also demonstrates the presence of secondary nucleation.^15^ Rodriguez Camargo et al. studied a longer tau segment without heparin and they showed that its aggregation is governed by a secondary nucleation once the initial aggregates are formed.^14^ The general trend in their kinetic data shows a longer and more pronounced lag phase, further indicating that heparin significantly accelerates the primary nucleation of tau. The largest oligomer of the Ac-PHF6-NH_2_ peptide (13-mer), found under the aggregating conditions in our present study, was detected after about 4.5 h, at a time point when many fibrils were already formed. From the IM-MS data presented in Figure 5, we see that the growth of isobaric oligomers with the same *m*/z 1580 continues at 24 h. This suggests that even when the ThT kinetic curve of the fibrils has reached the plateau, where ThT has attained its saturation binding, larger oligomers are still being formed and they continue to grow into fibrils. This is in good agreement with our CD data showing the change to classic β-sheet of the peptide sample after one month (Figure S3).

To get a deeper understanding of the role of heparin on aggregation, we compare the Ac-PHF6-NH_2_ peptide oligomers formed with heparin with our previous study focusing on oligomers of Ac-PHF6-NH_2_ peptide in heparin-free environment using the ESI-IM-MS approach.^36^ First, we see that heparin greatly accelerates fibril formation. Without heparin, fibril growth takes about 7 days, while with heparin fibril formation occurs within a few hours. This implies that heparin accelerates the typically slow primary nucleation process. However, even without heparin, a large variety of oligomers was observed in our previous work,^36^ but their appearance did not show any dynamic behavior in terms of new oligomers appearing over time. In the work presented here, the oligomers are very dynamic, with new oligomers appearing and disappearing over the course of 5 h. The major difference between our two studies is the use of nano-ESI here compared to normal ESI used in the previous study. First, nano-ESI is a significantly softer ionization method, requiring 2–3 times lower voltages compared to ESI, therefore making it possible to observe subtle changes over time. Second, the peptide concentration in the ESI study was 2.5 times higher than that used here. Finally, the smaller droplets in nano-ESI (typical size <0.6 μm)^50^ compared to ESI (3 μm) mean that these nano-ESI droplets also contain fewer peptide molecules per droplet, preventing additional clustering within the droplets, as was previously shown for the serine clusters.^51^ In order to probe the delicate aggregation dynamics of the oligomers nano-ESI should be employed, since ESI hides the dynamics of the peptide aggregation occurring in solution. As can be seen from the CCS values of the Ac-PHF6-NH_2_ peptide oligomers with and without heparin presented in Table S1, the CCS values are the same for most of the observed oligomers (within ∼2%). This suggests that heparin does not alter the structure of the oligomers that were observed in both experiments. The ability of heparin to align the Ac-PHF6-NH_2_ peptide oligomers to a parallel β-sheet to some extent was observed previously in simulations,^11^ however Infrared Multiple Photon Dissociation (IRMPD) spectroscopy would be required to observe more structural details.

In summary, we have studied the dynamics of aggregation of the Ac-PHF6-NH_2_ peptide from tau protein with heparin using TEM and CD to characterize the fibril morphology, ThT fluorescence assays to obtain kinetic data, and soft nano-ESI-IM-MS approach to investigate the structural properties of the formed oligomers. Heparin allowed us to precisely control the time course of tau aggregation and was used to accelerate aggregation. It was concluded that heparin can serve as a nucleation site for the tau segment, which follows a secondary nucleation mechanism. In addition, it appears that heparin does not alter the structure of the early-stage oligomers, based on their CCS values measured with and without heparin. From the IM-MS data and the derived CCS values, we have shown that the size of larger oligomers becomes compact starting from the hexamer, suggesting that as the oligomers increase in size, they become highly packed and adopt a multi-layered arrangement of β-sheets. It has also been shown that Aβ42 and TDP-43 peptides form a hexamer that adopts a β-sheet barrel conformation, which could also happen in our case with the Ac-PHF6-NH_2_ peptide.^52,53^ To investigate the β-sheet character of these oligomeric structures, a follow-up study using gas-phase IRMPD action spectroscopy on the newly developed Photo-Synapt should be performed.^54^ To elucidate the intermolecular interactions between tau peptide and heparin using the IM-MS approach, a completely new method should be developed focusing on heparin, and its ability to bind the tau peptide, as has been shown by calculations,^11^ which is rather challenging due to the polymeric nature of heparin and lies beyond the scope of the current work.

## Methods

### Materials and Peptide Samples Preparation

Ammonium acetate (5 M), Thioflavin T (ThT), 1,3,3,3-hexafluoro-2-propanol (HFIP), and heparin (H4784) were purchased from Sigma-Aldrich. The Ac-PHF6-NH_2_ peptide (Ac-^306^VQIVYK^311^–NH_2_) was purchased from Biomatik (>95% purity) and used without any further purification. Peptide aliquots were prepared using HFIP as described before.^36^ Briefly, 1 mg of peptide was dissolved in 1 mL of HFIP and sonicated for 5 min. 50 μL of this solution was pipetted into aliquots, which were dried in a fume hood for 3–12 h until the HFIP was completely evaporated. The aliquots containing the dried peptide were stored at −20 °C. All peptide solutions were prepared in 10 mM ammonium acetate (AA) diluted in Milli-Q water. The pH of the 10 mM AA solution was adjusted to pH 7.3–7.4 with a 0.5% ammonia/water solution.

### Sample Preparation for ThT Assays and Instrumental Parameters

The 6 mM ThT stock solution was prepared in Milli-Q water and filtered through a 0.2 μm syringe-driven filter unit (Millex). Ac-PHF6-NH_2_ peptide aliquots of 100 μM were prepared in 10 mM AA with 20 μM ThT. This solution was vortexed for a few seconds and further diluted to the desired peptide concentration of 25, 20, 15, and 12.5 μM. 100 μL of these Ac-PHF6-NH_2_ peptide samples were added to the 96 well plate (Corning, ref 3881). Every concentration was measured in triplicate. To initiate aggregation, 1 μL of heparin stock solution (150 μM in 10 mM AA) was added to each well, resulting in a final heparin concentration of 1.5 μM per well. The contents of each well were mixed using a multichannel pipette. The well plate was then sealed with a nontransparent film (Corning, ref 6570) and inserted into the plate reader (CLARIOstar Plus, BMG labtech). The excitation and emission wavelengths were set at 440 and 480 nm, respectively. Bottom detection was used, and readings were taken every 5 min. The temperature of the plate reader was set at 25 °C and the measurements were performed quiescently.

### Sample Preparation for TEM and Instrumental Parameters

Three μL of the Ac-PHF6-NH_2_ peptide sample (25 μM in 10 mM AA with 20 μM ThT and 1.5 μM heparin) was taken directly from the well plate and prepared for the TEM visualization 4 days after sample preparation. The peptide sample was applied to a freshly glow-discharged carbon-coated mesh grid (size 300). After allowing it to stand for 2 min and blotting off the excess liquid, the samples were contrasted with 2% uranylacetate in water for 40 s. Subsequently, excess stain was blotted off, and the grids were air-dried. Fibrillar structures were imaged on a 200 kV Talos F200X G2 (ThermoFisher) TEM using a 4k × 4k pixel Ceta 16 M camera.

### Sample Preparation for IM-MS and Experimental Parameters

The Ac-PHF6-NH_2_ peptide samples were diluted to 20 μM in 10 mM AA, which was used for all IM-MS experiments. Ten microliters of this sample was loaded into nano-ESI capillaries and measured immediately and after 24 h. The nano-ESI capillaries were made from the borosilicate capillaries (1.0 mm outer diameter, 0.75 mm inner diameter) pulled with a P-1000 micropipette puller (Sutter Instrument) and sputter coated with gold using a 108auto sputter coater (Cressington). Heparin stock solution was added to the same 20 μM of Ac-PHF6-NH_2_ peptide solution in 10 mM AA, resulting in a heparin concentration of 1.5 μM. This peptide sample with heparin was loaded simultaneously into 5–6 nano-ESI capillaries, which were then used for the time point measurements.

IM-MS experiments were performed on a TIMS-Qq-ToF^55−58^ (first generation) mass spectrometer (Bruker Daltonics GmbH). Peptide samples were introduced in positive mode using a home-built nano-ESI source based on the design from the group of Pagel.^59^ A set of soft instrumental parameters, based on those developed previously, was applied here to ensure minimal fragmentation of peptide oligomers during their transfer through all stages of the ion mobility mass spectrometer.^36,60^ A complete overview of the experimental settings can be found in Table S2 in the Supporting Information (SI). Ion mobility and mass spectra were calibrated internally in the DataAnalysis v5.2 software (2019 Bruker Daltonics GmbH). Briefly, a residual Agilent ESI Tuning Mix (*m*/*z* 622, 922, 1222, 1522) and additional abundant masses in each spectrum of *m*/*z* 462.1, 536.2, and 790.5 with the following inverse reduced ion mobility values of 0.985, 1.190, 1.382, 1.556, 0.948, 1.037, and 1.348 V·s/cm^2^ were used for the above *m*/*z* values. The mobility values of additional masses were obtained after external calibration using Tuning Mix.

## Data Availability

The data underlying this study are openly available in DataCite Commons at https://doi.org/10.48338/vu01-4jgimp.

## Supporting Information Available

Schematic workflow; AmyloFit models fitted for the data; CD spectrum of aggregated sample; additional IM and MS spectra; comparison of CCS values with and without heparin; kinetics of fibrils and oligomers, and instrumental parameters of IM-MS experiments (PDF)
