# Supplementary material for: Unraveling the Structure and Dynamics of Ac-PHF6-NH2 Tau Segment Oligomers
Source: ACS Chem Neurosci. 2024 Aug 31;15(18):3391–400. doi: 10.1021/acschemneuro.4c00404 (PMC11413852; doi:10.1021/acschemneuro.4c00404)
Supplement: Supplementary file 1 — cn4c00404_si_001.pdf [file cn4c00404_si_001.pdf]

Supporting Information

Unraveling the Structure and Dynamics of Ac-PHF6-NH_2 Tau Segment Oligomers

Iuliia Stroganova[1,2], Zenon Toprakcioglu[3], Hannah Willenberg[1,†], Tuomas P. J. Knowles[3,4], and Anouk M. Rijs[1,2,*]

1. Division of Bioanalytical Chemistry, Department of Chemistry and Pharmaceutical Sciences, Amsterdam Institute of Molecular and Life Sciences, Vrije Universiteit Amsterdam, De Boelelaan 1105, 1081 HV Amsterdam, the Netherlands.

2. Centre for Analytical Sciences Amsterdam, 1098 XH, Amsterdam, the Netherlands.

3. Centre for Misfolding Diseases, Yusuf Hamied Department of Chemistry, University of Cambridge, Cambridge CB2 1EW, United Kingdom.

4. Cavendish Laboratory, Department of Physics, University of Cambridge, Cambridge CB3 0HE, United Kingdom.

Correspondence to: Anouk M. Rijs, e-mail: a.m.rijs@vu.nl

Contents

Figure S1. Schematic workflow of the experiments to elucidate the aggregation kinetics of the Ac-PHF6-NH2 peptide in 10 mM AA with addition of heparin....2

Figure S2. Aggregation kinetics of Ac-PHF6-NH2 peptide in 10 mM AA with 20 µM ThT and 1.5 µM heparin at different peptide concentrations....2

Figure S3. CD spectrum of Ac-PHF6-NH2 peptide....3

Figure S4. Averaged mass spectra with and without heparin....3

Figure S5. Quadrupole-selected total ion mobility spectrum of m/z 1580 of Ac-PHF6-NH2 peptide....4

Table S1. Comparison of the CCS values of Ac-PHF6-NH_2 peptide measured on TIMS-Qq-ToF....4

Figure S6. CCS values versus oligomer number n....5

Figure S7. Normalized intensities of fibrils and oligomers....6

Table S2. Instrumental parameters on TIMS for IM-MS measurements....7

References....8

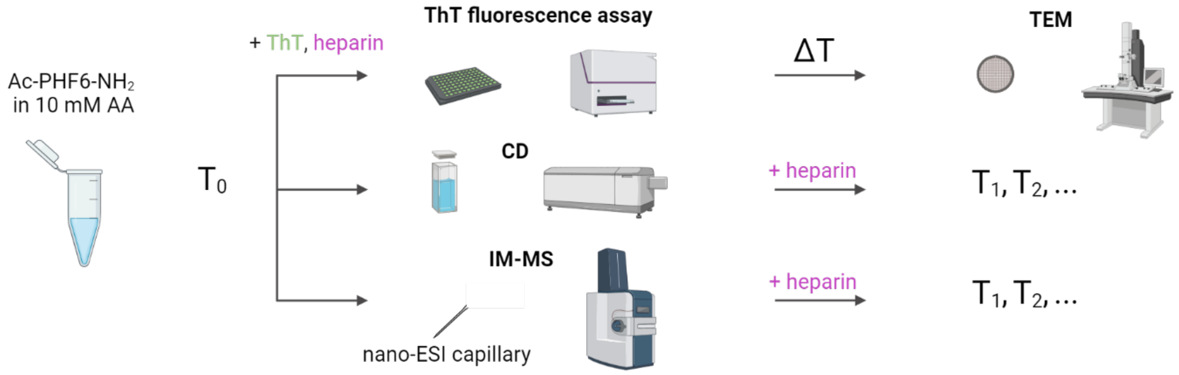

Figure S1. Schematic workflow of the experiments to elucidate the aggregation kinetics of the Ac-PHF6-NH2 peptide in 10 mM AA with addition of heparin. The peptide is measured without heparin for the initial time point T_0 by IM-MS and CD. To initiate assembly, heparin is added to the sample and the kinetic data is measured with the ThT assays. The sample is then removed to visualize the fibrils by TEM. The time points with heparin are measured by CD and IM-MS. Created with BioRender.com.

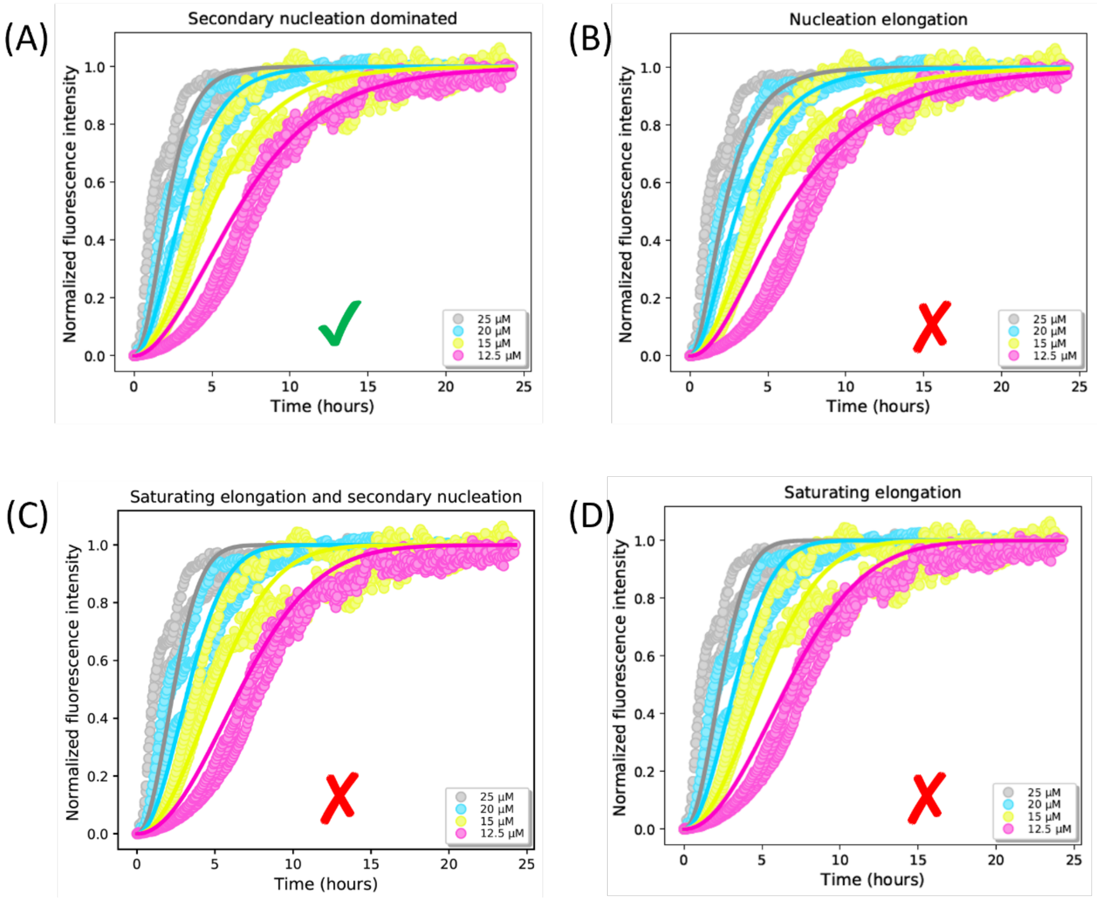

Figure S2. Aggregation kinetics of Ac-PHF6-NH2 peptide in 10 mM AA with 20 µM ThT and 1.5 µM heparin at different peptide concentrations: 25, 20, 15 and 12.5 µM (gray, blue, yellow, and pink, respectively). Each peptide concentration was measured in triplicate. The data were fitted (solid lines) using a global fit with $n_c = n_2 = 3$ and assuming a secondary nucleation model (A), nucleation elongation model (B), saturating elongation and secondary nucleation (C), and saturating elongation (D) in AmyloFit. The mean residuals errors are the following 0.00543 (A), 0.00571 (B), 0.00730 (C), and 0.00729 (D). The saturating elongation model as well as saturating elongation and secondary nucleation model both result in the highest error and describes 25 and 12.5 µM less good at the plateau phase than the secondary nucleation model.

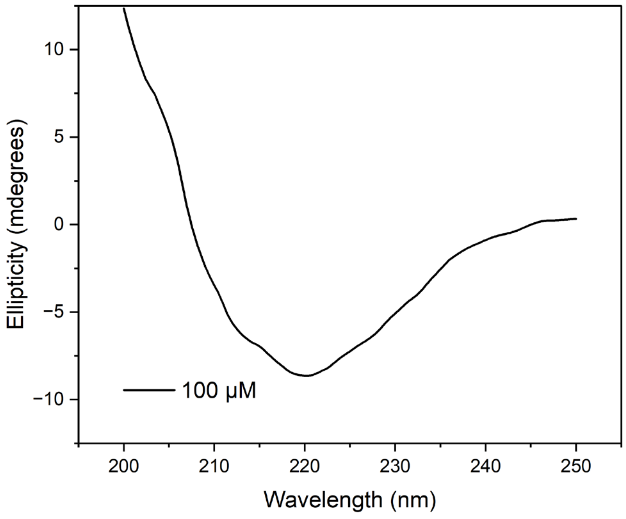

Figure S3. CD spectrum of Ac-PHF6-NH2 peptide 100 μM in 10 mM AA with 1.5 μM heparin and 20 μM ThT taken after incubation at room temperature after 33 days.

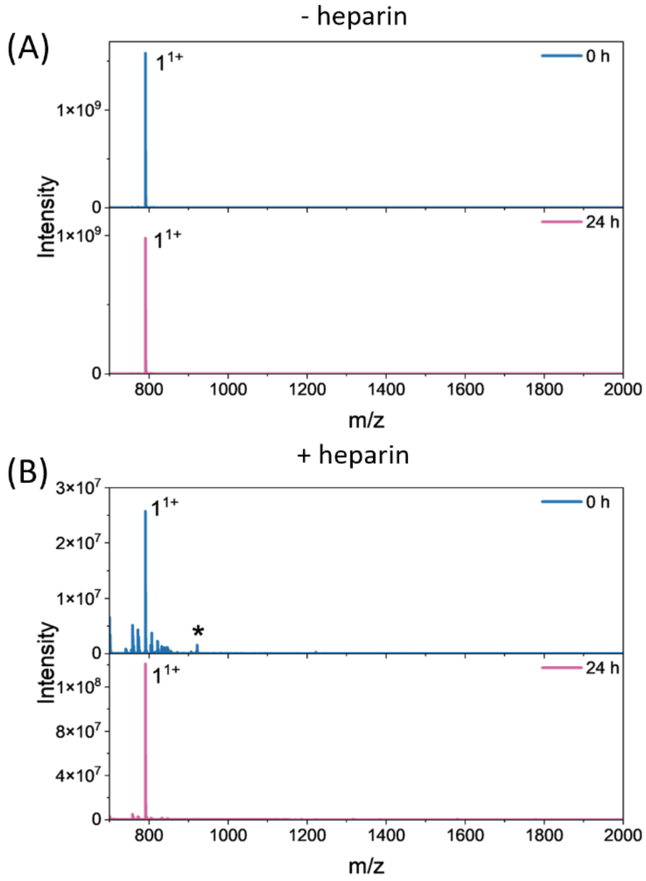

Figure S4. (A) Averaged mass spectra without heparin measured immediately after the sample preparation (blue) and after 24 hours (pink). (B) Averaged mass spectra with addition of heparin (final concentration 1.5 μM) measured immediately after addition of heparin (blue) and after 24 hours (pink). The main peak present is the singly charged monomer (*m/z* 790.5) denoted as 1^{1+} (n^{z+}), where n is number of monomers and z is the charge state. The asterisk corresponds to the TuningMix calibrant of *m/z* 922.

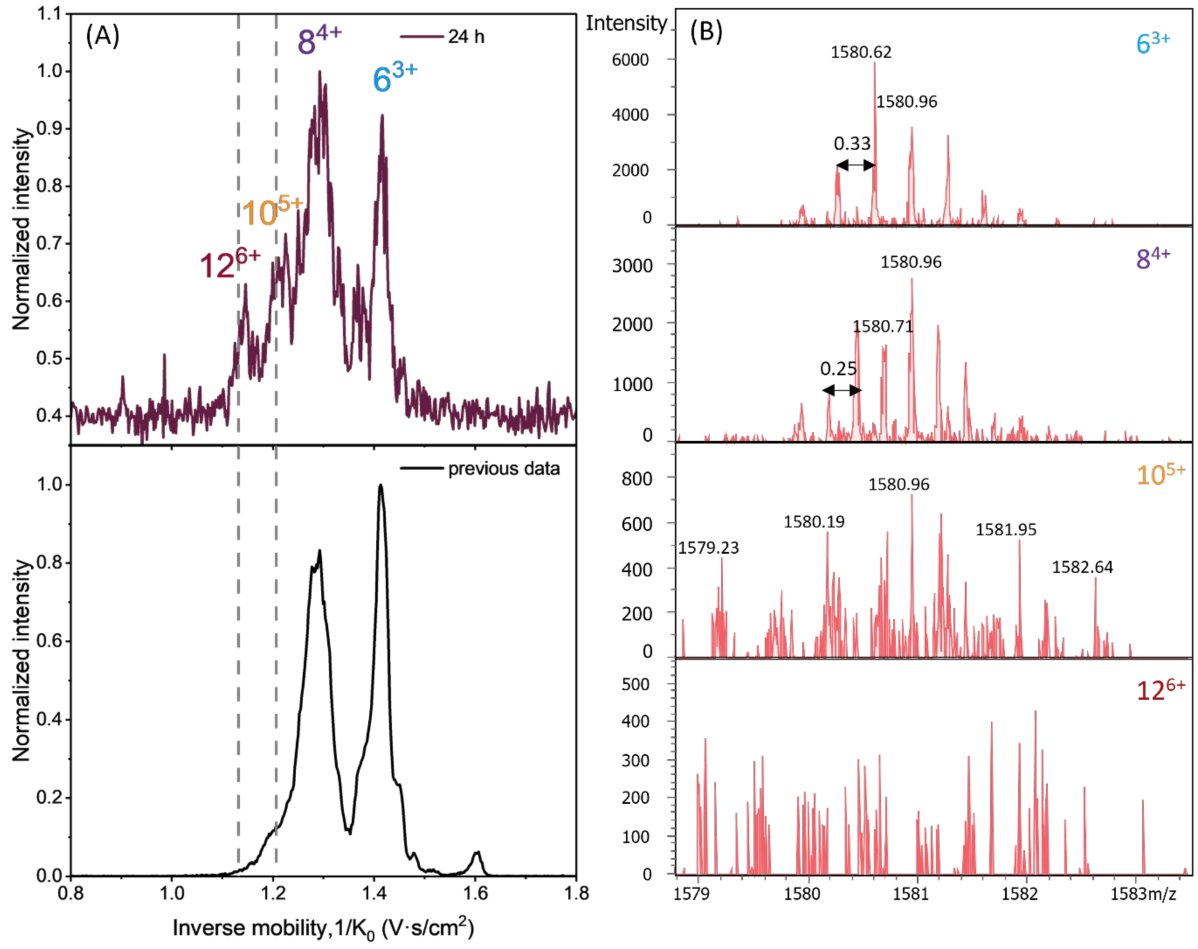

Figure S5. (A) Quadrupole-selected total ion mobility spectrum of m/z 1580 of 20 µM of Ac-PHF6-NH2 peptide in 10 mM AA with 1.5 µM heparin measured at 24 hours after heparin addition (shown in dark red, top). The oligomers are labeled as $[2n]^{nz+}$, where n is the number of monomers and z is the charge state. Quadrupole-selected total ion mobility spectrum of *m/z* 1580 of 50 µM of Ac-PHF6-NH_2 peptide in 10 mM AA, measured by us previously[1] (shown in black, bottom). The dashed gray lines show the position of the 10^{5+} and 12^{6+} oligomers previously assigned. The two left peaks from the upper spectrum overlap with these lines. (B) Extracted mass spectra from the ion mobility peaks corresponding to the oligomers showing the isotopic pattern. The spacing between the peaks indicates the triply-charged species 6^{3+} and quadruply-charged oligomer 8^{4+}, respectively.

Table S1. Comparison of the CCS values of Ac-PHF6-NH_2 peptide measured on TIMS-Qq-ToF.

Oligomer *m/z*, n^{z+}	^{TIMS}CCS, Å^2 from current work	^{TIMS}CCS, Å^2 from the previous data[1]	Relative difference, %
790.48, 1^{1+}	275	275	0.0
1185.22, 3^{2+}	574	573	0.2
1316.79, 5^{3+}	793	792	0.1
1579.95, 6^{3+}	858	857	0.1
1382.59, 7^{4+}	997	1000*	0.3
1579.95, 8^{4+}	1066	1049	1.6
1777.33, 9^{4+}	1099	1108*	0.8
1974.69, 10^{4+}	1145	1146	0.1

*The CCS values in blue indicate the oligomers, which were assigned to two conformers in the previous work. In the current work, the signal was too noisy to distinguish between conformers, therefore the closest CCS value from the previous work is compared to the current CCS value.

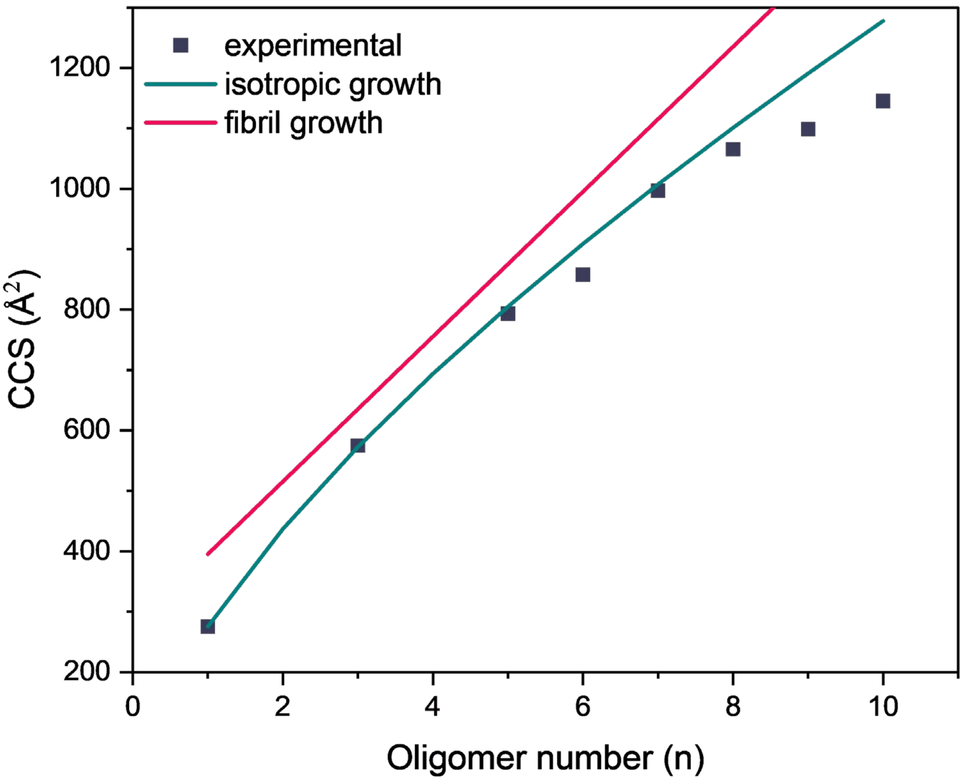

Figure S6. Collision cross section (CCS) values versus oligomer number n (shown in black), where n is the number of monomer units in the oligomer, for the Ac-PHF6-NH_2 peptide in 10 mM AA with 1.5 µM heparin. The green line corresponds to the isotropic growth[2], where the CCS values scale as $\sigma_1 \cdot n^{2/3}$ (n is the number of monomers in the oligomer and σ_1 is the CCS value of the monomer). The red line corresponds to the stacked fibrillar growth[2] in one direction, where the CCS values has the following linear dependence on n: $\sigma_1 = 120 \cdot n + 275$. Here, the slope a = 120 is the difference of CCS values between dimer (2^{1+}) and monomer (1^{1+}), and the intercept k = 275 is the CCS value of monomer (1^{1+}).

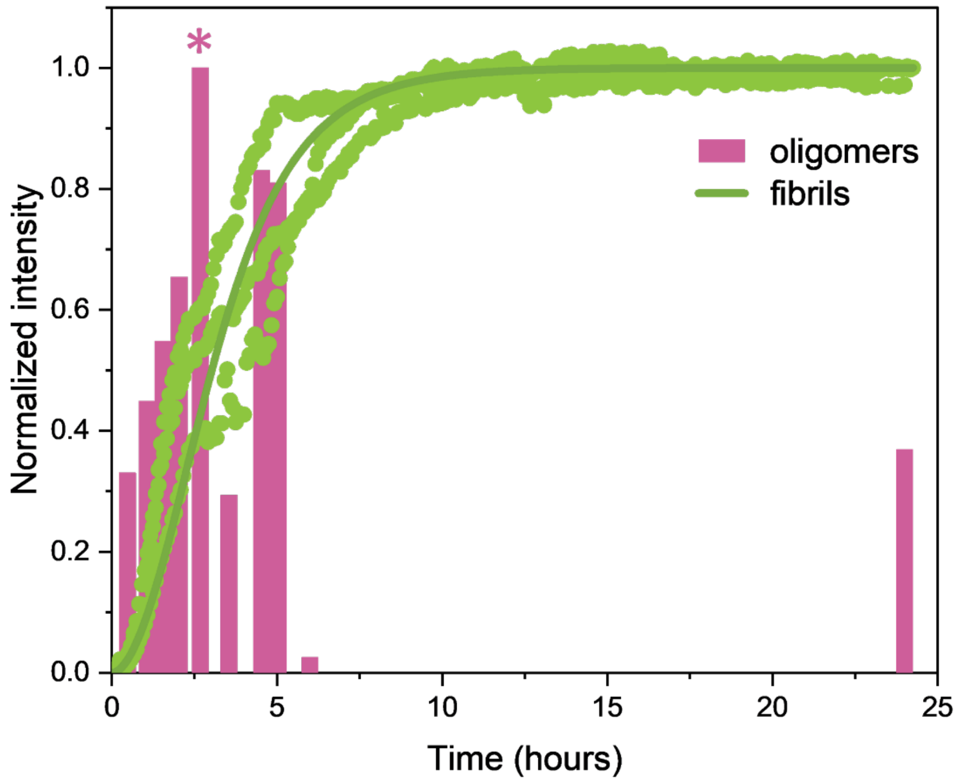

Figure S7. Normalized intensities of fibrils (green) and oligomers (pink) of 20 µM of Ac-PHF6-NH_2 peptide in 10 mM AA with 1.5 µM heparin over time. The pink asterisk indicates the highest oligomer intensity. The solid green line shows the continuing growth of fibrillar species.

Table S2. Instrumental parameters on TIMS for IM-MS measurements.

Source	
**Capillary voltage**	*900-1200 V*
**End Plate offset**	*200 V*
**Nebulizer pressure**	*0.1 Bar*
**Dry gas flow**	*2 L/min*
**Dry temperature**	*80°C*
**General parameters**	
**Funnel 2 RF**	*100 Vpp*
**Multipole RF**	*50 Vpp*
**isCID energy**	*0 eV*
**Deflection delta**	*0 V*
**Low mass**	*300 m/z*
**Ion energy**	*10 eV*
**Collision energy**	*1 eV*
**Collision RF**	*1300 Vpp*
**Transfer time**	*90 µs*
**Pre Pulse storage**	*14 µs*
**Mass range**	*50-3000*
**Mode**	*positive*
**IMS parameters**	
**D1**	*0 V*
**D2**	*10 V*
**D3**	*20 V*
**D4**	*20 V*
**D5**	*0 V*
**D6**	*20 V*
**Funnel 1 RF**	*250 Vpp*
**Collision Cell In**	*200 V*
**Accumulation time**	*2 ms*
**Ramp time**	*100 ms*
**IMS range**	*0.50-1.60* $V\cdot s/cm^2$
**TIMS Tunnel In pressure**	*2.297-2.312 mbar*

References:

(1) Stroganova, I.; Willenberg, H.; Tente, T.; Depraz Depland, A.; Bakels, S.; Rijs, A. M. Exploring the Aggregation Propensity of PHF6 Peptide Segments of the Tau Protein Using Ion Mobility Mass Spectrometry Techniques. *Anal. Chem.* **2024**. https://doi.org/10.1021/acs.analchem.3c04974.

(2) Bleiholder, C.; Dupuis, N. F.; Wyttenbach, T.; Bowers, M. T. Ion Mobility-Mass Spectrometry Reveals a Conformational Conversion from Random Assembly to β-Sheet in Amyloid Fibril For-Mation. *Nat. Chem.* **2011**, *3*. https://doi.org/10.1038/nchem.945.
